# Anti-cancer effect of *Scutellaria baicalensis* in combination with cisplatin in human ovarian cancer cell

**DOI:** 10.1186/s12906-017-1776-2

**Published:** 2017-05-25

**Authors:** Bo Yoon Choi, Jong Cheon Joo, Yeon Kyu Lee, Ik-Soon Jang, Soo Jung Park, Yoon Jung Park

**Affiliations:** 1 Department of Nutritional Science and Food Management, College of Science & Industry Convergence, Ewha Womans University, Seoul, Republic of Korea; 2 Department of Sasang Constitutional Medicine, College of Korean Medicine, Wonkwang University, Iksan, Republic of Korea; 3 Department of Bioconvergence, Korea basic science institute, Daejeon, Republic of Korea; 4 Department of Sasang Constitutional Medicine, College of Korean Medicine, Woosuk University, Jeonju, Republic of Korea

**Keywords:** Cell death, Drug resistance, Epigenomics, Herbal medicine, Ovary Neoplasms

## Abstract

**Background:**

Ovarian cancer is one of the major causes of death among females in worldwide. Cisplatin is a primary anti-cancer drug against ovarian cancer, but the recurrent tumors after treatment frequently show acquired chemoresistance. Extract of *Scutellaria baicalensis* (SbE) has been reported to have functional compounds including baicalin, which has anti-cancer effects. However, the anti-cancer effects of SbE in ovarian cancer and its underlying mechanisms are elusive.

**Methods:**

We investigated that the effects of SbE and/or cisplatin on cell death in the cisplatin sensitive ovarian cancer cell line A2780 (CSC) and the counterpart cell line that has cisplatin resistance (CRC). Molecular mechanisms of the effects, focusing on apoptosis and autophagy, were examined.

**Results:**

Treatment of cisplatin or SbE reduced cell viability significantly in CSC and too much lesser extent in CRC. Cisplatin-induced cell death in CSC was mediated by p53-induced apoptosis acompanied by expresson of damage-regulated autophagy modulator (*DRAM*). In CRC, decreased *DRAM* expression (*p* < 0.01) hindered p21-mediated cell death and contributed to cisplatin resistance. Treatment of SbE also induced cell death in CSC by p53-dependent apoptosis, not in CRC. Autophagy was not induced by neither cisplatin nor SbE. Intriguingly, the combinational treatment of SbE and cisplatin significantly decreased cell viability in CRC. The cell death was mediated by autophagy with increased expression of *Atg5* and *Atg12* (*p* < 0.05), rather than p53-dependent pathway with repressed expression of *p21* (*p* < 0.001) through HDAC1 activation.

**Conclusions:**

The combined treatment of SbE with cisplatin was effective in CRC, leading to cell death via Beclin1-independent autophagy, suggesting that SbE treatment in combination with cisplatin has a potential as a chemotherapeutic agent in cisplatin-resistant ovarian cancer.

## Background

Ovarian cancer has remained one of common and lethal gynecological cancers for women in worldwide [1]. About the 70% of ovarian cancers are diagnosed at advanced stage [2] and a five-year survival rate was recorded about the 40% in all cancer staging of ovarian cancer [3]. Although therapeutic methods against cancer such as chemotherapy and surgery have rapidly developed in the past decades, identification of successful treatments against the ovarian cancer has been challenged due to its high rate for late-stage diagnosis and acquisition of drug resistance.

Cisplatin is a commonly used drug in the treatment of ovarian cancer, but it often faces the challenge of chemoresistance after repeated treatments, resulting in limiting drug effectiveness [4]. The resistance can be caused by multiple mechanisms, including inadequate cisplatin accumulation, cisplatin inactivation, enhanced DNA repair, and activation of survival signaling pathways [5]. Previous studies have found that expression level of the genes, related to intracellular drug accumulation, drug inactivation, DNA repair system, and survival signaling pathway, was significantly different between CSC and CRC [6–9].

Drug resistant cancer cells show uncontrolled cell proliferation and reduced cell death such as apoptosis and autophagy not responding to the treatment [10]. Apoptosis is a programmed process of unnecessary or dysfunctional cell death via DNA fragmentation, nuclear fragmentation, chromatin condensation, membrane blebbing and cell shrinkage [11]. p53 is a key modulator of cellular stress responses. p53 triggers apoptosis through up-regulating target genes in many cell types including cancer cells [12]. Target genes induced by p53 include the cyclin-dependent kinase inhibitor *p21* gene and the pro-apoptotic *Bax* gene leading to apoptosis [13–15]. *DRAM* gene is another target gene, which is an important component of p53-induced apoptosis and triggers autophagy [16]. Autophagy is an intracellular self degradative process that dismantles unnecessary or dysfunctional cytoplasmic components and organelles in the lysosome. In cancer cells, some of anti-cancer therapeutic agents promote autophagy-induced cell death [17]. Autophagic pathway occurs through the formation of double membrane vesicle called autophagosome that encloses cytoplasmic components and organelles and then autophagosome transfers to lysosome for degradation [17]. Autophagosome formation involves multiple factors such as Beclin 1, autophagy-related protein (Atg)12-Atg5, and microtubule-associated protein light chain 3 (LC3) complexes [18]. The transfer to lysosome also requires DRAM in its membrane [19].

Extract of *Scutellaria baicalensis* (SbE) is an herbal medicine that have been used for anti-oxidant and anti-inflammatory activities [20]. It is known to have multiple functional compounds including baicalin and baicalein. Baicalin is a flavone glycoside that has been reported to have anti-cancer effects in breast cancer and prostate cancer [21, 22]. Although baicalin as a single compound has been studied for its anti-cancer properties, few studies are available for anti-cancer effects of the extract [23]. In this study, we investigated whether SbE contributed to overcome cisplatin resistance using a cisplatin-resistant ovarian cancer cell model and its possible mechanisms.

## Methods

### Preparation of SbE

Lyophilized SbE was obtained from Hanpoong Pham & Foods Co., Ltd. (Jeonju, Korea). 300 g SbE was refluxed for 3 h in 3 L of 30% ethanol, passed through 1 μm filter, evaporated, and dried in vacuum less than 60 °C and pulverized. SbE, obtained with 115.3 g (38.43% yield), was dissolved in dimethyl sulfoxide (DMSO) to make stock solutions of 250 mg/mL and then was diluted with serum-free RPMI 1640 for the working concentrations (100 ~ 400 μg/mL), resulting in the percentage of DMSO to dissolve the extract was less than 0.16%, in final. Equal amounts of DMSO were included in controls.

### Liquid chromatography-mass spectrometer (LC-MS) analysis

A liquid chromatograpy mass spectroscopy (LC-MS) analysis was achieved using an Agilent 6410B triple quadrupole (Agilent Technologies, Wilmington, DE, USA) equipped with electrospray ionization (ESI) (Agilent Technologies, Wilmington, DE, USA), according to a manufacturer’s protocol. Briefly, 100 mg sample dissolved in 1 mL of MeOH and centrifuged. Volume of sample injection into HPLC system (1200 Series LC, Agilent Technologies, Wilmington, DE, USA) was 5 μL. 150 cm × 2 mm^2^, 4 μm Synergi Hydro-RP 80 Å column (Phenomenex, Torrance, CA, USA) was used for LC separation at 30 °C. ESI activated at 3 kV and 380 °C as a source temperature. LC-ESI-MS was measured under the following conditions: capillary voltage = 3 kV, cone voltage = 30 kV, source offset = 30 V, nebulizer pressure = 15 bar, desolvation gas flow-rate = 650 L/h, cone gas flow-rate = 150 L/h, fragmentor voltage = 90 V, collision voltage = 20 V. 0.1% formic acid in distilled water as mobile phase A and 0.1% formic acid in acetonitrile as mobile phase B separated the sample and went into the ESI chamber at a flow rate of 0.5 mL/min for 20 min. Sample was detected by multiple-reaction monitoring mode (MRM) of monitoring the transition pairs at m/z 252.1/136.1.

### Cell culture

The cisplatin sensitive ovarian cancer cell lines (CSC) A2780 and the cisplatin resistant cell lines (CRC) A2780cis were obtained from Dr. Jung-Hyuck Ahn (Ewha Womans University school of medicine, Seoul, Korea). A2780 and A2780cis cells were cultured in RPMI 1640 (Welgene, Daegu, South Korea) supplemented with 10% fetal bovine serum (FBS) (Atlas, Fort Collins, CO, USA), 1% penicillin/streptomycine (Gibco, Gaithersberg, MD, USA) in a humidified atmosphere of 5% CO_2_ at 37 °C. A2780cis cells were supplemented 100 μM of cisplatin (sigma, St. Louis, MO, USA) in medium every even cell passage. To investigate anti-cancer effects of SbE, cells were cultured in RPMI 1640 supplemented with 10% FBS and 1% penicillin/streptomycine. After 24 h, 100 ~ 400 μg/mL of SbE and/or 10 ~ 100 μM of cisplatin or 28 ~ 56 μM of baicalin diluted in serum free RPMI 1640 were treated the cells for 24 h.

### MTT assays

Cell viability was measured by MTT assays [24]. 1X10^4^ cells per well were seeded in 96-well plates and incubated at 37 °C. After 24 h, a range of concentrations of SbE and/or cisplatin or baicalin were treated to wells and incubated at 37 °C for 24 h. After 22 h treatment of SbE and/or cisplatin or baicalin, 3-[4,5-dimethylthiazol-2-yl]-2,5-diphenyltetrazoliumbromide (MTT) (Sigma Aldrich, St, Louis MO, USA) solution like one-tenth the original culture volume was added in the treated cells for 2 h. MTT solution was dissolved in phosphate buffered saline (PBS) to make stock solution 5 mg/mL. Then DMSO was added to convert MTT to purple formazan in mitochondria of viable cells. Microplate reader (Biochrom, Berlin, Germany) read viable cells using the absorbance of 562 nm.

### RNA isolation and reverse transcription

A2780 and A2780cis cells were harvested after treated with various concentrations of SbE and/or cisplatin or baicalin for 24 h. Total RNA was extracted using the trizol reagent (Life Technologies, Gaithersburg, MD, USA) and isopropanol precipitation. The pellets were dissolved in Tris-EDTA (TE) buffer. 500 ng RNA was used for complementary DNA (cDNA) synthesis using RevertAid reverse transcriptase (Thermo Scientific, Waltham, MA, USA), according to the manufacturer’s protocol.

### Quantitative reverse transcriptase (qRT)-PCR

cDNA was used for qRT-PCR to investigate gene expression levels. qRT-PCR was performed with SYBR Green PCR Master mix (Qiagen, Hilden, Germany) using PCR machine, Rotor-Gene Q machine (Qiagen, Hilden, Germany). The primer pairs were the followings: for *Atg5*, 5′-TGGAGTAGGTTTGGCTTTGG-3′ and 5′- ATGGTTCTGCTTCCCTTTCA-3′, for *Atg12*, 5′-CCTTTGCTCCTTCCCCAGA-3′ and 5′-ATCCCCACGCCTGAGACTT-3′, for *Bax*, 5′-CGTGGACACAGACTCCCC-3′ and 5′-CCAATGTCCAGCCCATGATG-3′, for *Beclin 1*, 5′-ACCAACGTCTTTAATGCAACCT-3′ and 5′- CATGGAGCAGCAACACAGTC-3′, for *DRAM*, 5′-CATCCCCATGATTGTCTGTG-3′ and 5′-AAAGGCCACTGTCCATTCAC-3′, for *HDAC1*, 5′- GGTCTCTACCGAAAAATGGAAA-3′ and 5′-TTGCTGTACTCCGACATGTTATC-3′, for *p21*, 5′-TGTCTTGTACCCTTGTGCCT-3′ and 5′- GGCGTTTGGAGTGGTAGAAA-3′, for *p53*, 5′-GCTGCTCAGATAGCGATGGT-3′ and 5′-CACGCACCTCAAAGCTGTTC-3′, and for *TBP*, 5′-AGCCAAGAGTGAAGAACAGTCC-3′ and 5′-CACAGCTCCCCACCATATTC-3′. Amplification was done at 95 °C for 5 min, followed by 40 cycles at 95 °C for 5 s and at 60 °C for 10 s. The relative expression of each gene of interest was calculated by normalization against TATA-box binding protein (TBP) expression levels in each sample.

### Statistical analysis

Results of cell viability and mRNA expression levels were indicated as mean ± standard deviation. The results were analyzed using two tailed Student’s t-test using Microsoft Excel 2010 (Microsoft, Redmond, WA, USA) and one-way analysis of variance (ANOVA) followed by Duncan post hoc test using SAS 9.4 (SAS Inc., Cary, NC, USA). *P* < 0.05 was considered to indicate a statistical significance in all experiments.

## Results

### Ovarian cancer cell models differently respond to cisplatin and cisplatin treatment induces p53-dependent apoptosis in CSC, not in CRC

**Fig. 1:**
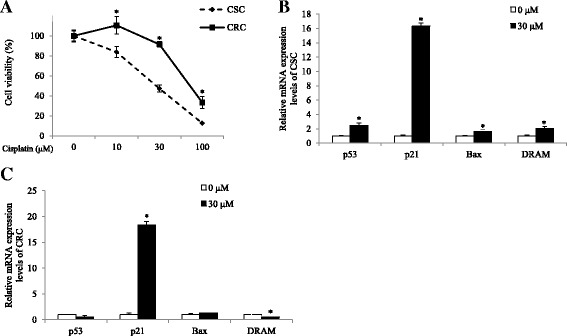
Effects of cisplatin on the cell viability and mRNA expression levels of *p53*, *p21*, *Bax*, and *DRAM* in CSC and CRC. Cells were exposed 10 ~ 100 μM of cisplatin for 24 h. **a** Cell viability according to cisplatin treatment in CSC and CRC was measured by MTT assay. After cells were exposed 30 μM of cisplatin for 24 h, mRNA expression levels of (**b**). *p53*, *p21*, *Bax* **,** and *DRAM* in CSC and (**c**). *p53*, *p21*, *Bax*, and *DRAM* in CRC were quantified by qPCR. All mRNA expression levels were normalized against *TBP*. Each value is the mean ± SD. *p* < 0.05 was taken to define statistical significance. Two tailed Student’s t-test and one-way ANOVA followed by Duncan post hoc test

To investigate the mechanisms underlying cisplatin resistance, we used a pair of ovarian cancer cell lines; the cisplatin sensitive A2780 as CSC and its counterpart that acquires the resistance as CRC. We firstly confirmed that CRC was less sensitive to cisplatin, compared to CSC. The response to cisplatin was measured by cell viability using an MTT assay. CSC and CRC were treated with from 10 μM to 100 μM of cisplatin for 24 h. As shown in Fig. 1a, cisplatin treatment decreased cell viability and the response to cisplatin in CRC was significantly lower than that in CSC. Cell viability in CRC was significantly higher than it in CSC in 10 μM, 30 μM, and 100 μM of cisplatin-treated groups (*p* < 0.001). Cell viabilites in CSC decreased to 84%, 47%, and 13% by 10 μM, 30 μM, and 100 μM of cisplatin treatment, respectively, compared with the non-treated group, while those in CRC decreased to 110%, 91%, and 33%. The difference of response to cisplatin between CSC and CRC was greater in the 30 μM of cisplatin-treated group than in the 10 μM or 100 μM of cisplatin-treated groups. Therefore, we used 30 μM of cisplatin treatment for further experiments.

We examined whether the difference in the induction of p53-mediated apoptotic pathway could explain the cisplatin resistance-involving mechanism in CRC. Expression levels of *p53*, *p21*, *Bax*, and *DRAM* genes were measured in the cells treated with 30 μM of cisplatin for 24 h. As shown in Fig. 1b, mRNA expression levels of pro-apoptotic *p53* (*p* < 0.05) and its target genes such as *p21* (*p* < 0.001), and *Bax* (*p* < 0.05), and *DRAM* (*p* < 0.05) significantly increased in the cisplatin-treated group, compared with the non-treated group in CSC, indicating that the cell death of CSC with cisplatin treatment invovled apoptosis through the p53-dependent pathway. On the other hand, CRC did not show significant changes in the expression of *p53* and *Bax* (Fig. 1c). Although mRNA levels of *p21* significantly increased, expression of *DRAM* rather decreased upon cisplatin treatment (*p* < 0.001; Fig. 1c). The results suggested that cisplitin treatment failed to induce apoptosis in the CRC, unlike CSC, due to the decreased expression level of *DRAM* gene, which is required for activation of the p53-dependent pathway.

### Cisplatin-induced cell death is not mediated by autophagy in ovarian cancer models

**Fig. 2:**
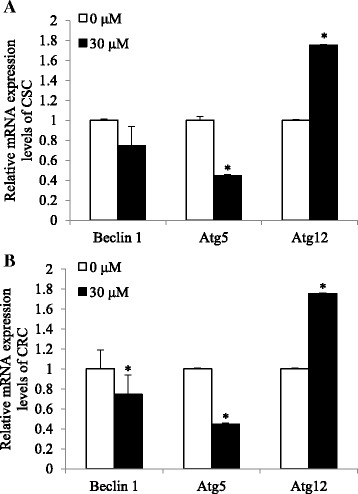
Effects of cisplatin on mRNA expression levels of *Beclin 1*, *Atg5*, and *Atg12* in CSC and CRC. After cells were exposed 30 μM of cisplatin for 24 h, mRNA expression levels of (**a**). *Beclin 1*, *Atg5*, and *Atg12* in CSC and (**b**). *Beclin 1*, *Atg5*, and *Atg12* in CRC were quantified by qPCR. All mRNA expression levels were normalized against *TBP*. Each value is the mean ± SD. *p* < 0.05 was taken to define statistical significance. One-way ANOVA followed by Duncan post hoc test

We investigated whether cisplatin-induced cell death is also mediated by autophagy. CSC and CRC were treated with 30 μM of cisplatin for 24 h and mRNA levels of autophay genes, *Beclin 1*, *Atg12*, and *Atg5* genes were measured. In CSC, mRNA expression levels of *Beclin 1* and *Atg5* did not significantly alter or decreased, respectively, while those of *Atg12* increased in cisplatin-treated group compared with the non-treated group (*p* < 0.01; Fig. 2a). Similarily, in CRC, mRNA expression levels of *Beclin 1* and *Atg5* decreased, while those of *Atg12* increased upon cisplatin treatment (*p* < 0.01; Fig. 2b). The results suggest cisplatin-induced cell death in CSC is not mediated by autophagy, since *Atg5* induction, which is esstial for the conjugation with Atg12 in autophagy, was not complete. In CRC, neither the p53-mediated apoptosis nor autophagy did not taken place, indicating the resistance against cisplatin.

### SbE is more effective to decrease cell viability than baicalin both in CSC and CRC

**Fig. 3:**
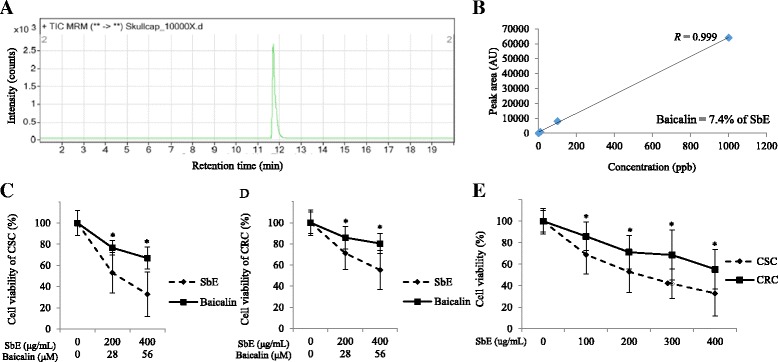
Quantitative analysis of baicalin in SbE and effects of SbE and baicalin on the cell viability in CSC and CRC. **a** LC-MS analysis of baicalin in SbE. **b** Baicalin peak area in LC-MS as a function of baicalin concentration (correlation coefficient, *R* = 0.999) and the percent content of baicalin in SbE. Cells were exposed 200 ~ 400 μg/mL of SbE or 28 ~ 56 μM of baicalin for 24 h. Cell viability according to SbE or baicalin treatment in (**c**) CSC and (**d**) CRC was determined by MTT assay. Cells were exposed 100 ~ 400 μg/mL of SbE for 24 h. **e** Cell viability according to various concentrations of SbE treatment in CSC and CRC was determined by MTT assay. Each value is the mean ± SD. *p* < 0.05 was taken to define statistical significance. Two tailed Student’s t-test

Next, we investigated whether SbE induced cell death in CSC and CRC to test a potential of SbE as an anti-cancer agent. We first analyzed quantitatively baicalin content in SbE using LC-MS analysis. The baicalin is a major flavonoid in SbE and has been reported for its effects on various cancer cells to inhibit cell proliferation and to induce cell death [21]. The quantity of baicalin in SbE was measured (Fig. 3a) and calculated according to a standard curve (Fig. 3b). As a result, we found that the content of baicalin in SbE was 74 mg/g (7.4%). Next, we investigated whether SbE and baicalin induced cell death in CSC and CRC at the various concentrations using an MTT assay. First, the cell lines were treated with from 200 μg/mL to 400 μg/mL of SbE and from 28 μM to 56 μM of baicalin, i.e. equivalent to the amounts in the extract, for 24 h to compre the effect of the extract and the single compound. As shown in Fig. 3c and d, both SbE and baicalin treatment decreased cell viability, however, the response of the SbE was significantly greater than that of baicalin. In CSC, cell viability in 200 μg/mL and 400 μg/mL of SbE-treated groups were significantly lower than it in 28 μM and 56 μM of baicalin-treated groups, respectively (*p* < 0.01 and *p* < 0.001, respectively). Likewise, in CRC, 200 μg/mL and 400 μg/mL of SbE treatment were significantly decreased compared to 28 μM and 56 μM of baicalin treatment, respectively (*p* < 0.05 and *p* < 0.01, respectively). Cell viability in CSC decreased to 53% and 33% by 200 μg/mL and 400 μg/mL of SbE treatment, respectively, compared with the non-treated group (Fig. 3c), while thoes in CRC decreased to 71% and 55% (Fig. 3d). On the other hand, cell viability in CSC decreased to 77% and 67% by 28 μM and 56 μM of baicalin treatment, respectively (Fig. 3c), while those in CRC decreased to 86% and 80% (Fig. 3d). Because the effects on cell viability of the extract is greater than that of single compound, baicalin, we focused on the effect of SbE for a subsequent experiments. We tested the effect of SbE on cell viability in more various concentrations. It effectively decreased cell viability in a dose-dependent manner in CSC and CRC and the response to SbE in CRC was significantly lower than in CSC (Fig. 3e).

### SbE treatment induces p53-dependent apoptosis in CSC, not in CRC

**Fig. 4:**
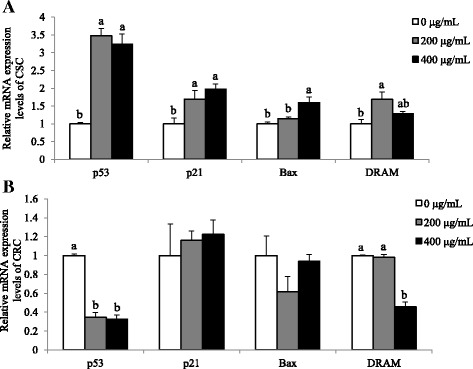
Effects of SbE on mRNA expression levels of *p53*, *p21*, *Bax*, and *DRAM* in CSC and CRC. After cells were exposed 200 ~ 400 μg/mL of SbE for 24 h, mRNA expression levels of (**a**). *p53*, *p21*, *Bax*, and *DRAM* in CSC and (**b**) *p53*, *p21*, *Bax*, and *DRAM* in CRC were quantified by qPCR. All mRNA expression levels were normalized against *TBP*. Each value is the mean ± SD. *p* < 0.05 was taken to define statistical significance. One-way ANOVA followed by Duncan post hoc test

We investigated by which mechanism SbE induced the cell death. Firstly, we tested whether SbE induced p53-mediated apoptotic pathway and its effect was similar in CSC and CRC. Expression level of p53-mediated apoptotic pathway such as *p53*, *p21*, *Bax*, and *DRAM* genes were measured in the cells treated with 200 ~ 400 μg/mL of SbE for 24 h. In CSC, mRNA expression levels of *p53* (*p* < 0.01), *p21* (*p* < 0.05), *Bax* (*p* < 0.05), and *DRAM* (*p* < 0.05) significantly increased in SbE-treated group, compared with the non-treated group (Fig. 4a). On the contrary, CRC showed that mRNA expression levels of *p53* and *DRAM* decreased (*p* < 0.001) and those of *p21* and *Bax* did not significantly alter after SbE treatment (Fig. 4b). The data suggested that SbE treatment induced apoptosis in the CSC, at least in part, via p53 pathway, but not in the CSC.

### SbE treatment induces autophagy in CSC, not in CRC

**Fig. 5:**
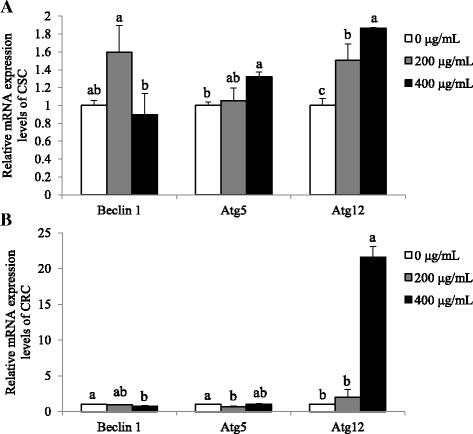
Effects of of SbE on mRNA expression levels of *Beclin 1*, *Atg5*, and *Atg12* in CSC and CRC. After cells were exposed 200 ~ 400 μg/mL of SbE for 24 h, mRNA expression levels of (**a**) *Beclin 1*, *Atg5*, and *Atg12* in CSC and (**b**) *Beclin 1*, *Atg5*, and *Atg12* in CRC were quantified by qPCR. All mRNA expression levels were normalized against *TBP*. Each value is the mean ± SD. *p* < 0.05 was taken to define statistical significance. One-way ANOVA followed by Duncan post hoc test

We investigated whether SbE-induced cell death also involved the autophagy mechanism. Expression levels of autophagy genes, *Beclin 1*, *Atg12*, and *Atg5* were measured in the cells treated with 200 ~ 400 μg/mL of SbE for 24 h. In CSC, mRNA expression levels of *Beclin 1* did not significantly alter, while those of *Atg5* (*p* < 0.05) and *Atg12* (*p* < 0.01) significantly increased in the SbE-treated group compared with the non-treated group (Fig. 5a). CRC, in contrast to CSC, showed that mRNA expression levels of *Beclin 1* and *Atg5* decreased (*p* < 0.05) after SbE treatment, while those of *Atg12* increased (*p* < 0.001) in the SbE-treated group compared with the non-treated group (Fig. 5b). As similar to the response to cisplatin, the autophagy-mediated cell death was induced in CSC, but not in CRC. The results proposed that SbE could not induce cell death neither p53-mediated apoptotic pathway or autophagy and its mechanism is involved in the resistance in CRC.

### CSC and CRC similarly respond to SbE combined with cisplatin

**Fig. 6:**
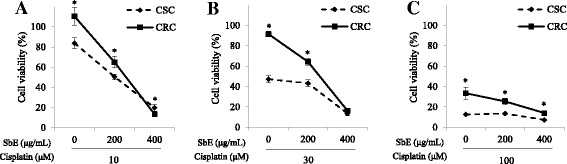
Combination effects of SbE and cisplatin on the cell viability in CSC and CRC. After cells were exposed 200 ~ 400 μg/mL of SbE and 30 μM of cisplatin for 24 h. Cell viability according to the combination of SbE and (**a**) 10 μM, (**b**) 30 μM, and (**c**) 100 μM of cisplatin treatment in CSC and CRC was measured by MTT assay. Each value is the mean ± SD. *p* < 0.05 was taken to define statistical significance. Two tailed Student’s t-test

Next, we tested whether a combined treatment of SbE and cisplatin had additive or synergic effects in CSC and CRC. CSC and CRC were treated with 200 ~ 400 μg/mL of SbE and 10 μM ~ 100 μM of cisplatin for 24 h. The response to the combined treatment in CRC was as sensitive as that in CSC by using MTT assays (Fig. 6). When the ovarian cancer cells were treated with the combined treatment, the cell viability between CSC and CRC gradually became closer depending on the concentration of the SbE at 30 uM of cisplatin, where the viability showed maximal difference between CSC and CRC upon only cisplatin treatment (Fig. 1a). The cell viability in CSC decreased to 47%, 43%, and 14%, while those in CRC decreased to 91%, 64%, and 16% by 0 μg/mL, 200 μg/mL, and 400 μg/mL of SbE with 30 μM of cisplatin compared with the non-treated group (Fig. 6b). The data show that the combined treatment of SbE and cisplatin has a potential as a chemotherapeutic method to overcome chemoresistance.

### The treatment of SbE, combined with cisplatin, induces not apoptotic but autophagic cell death in CRC

**Fig. 7:**
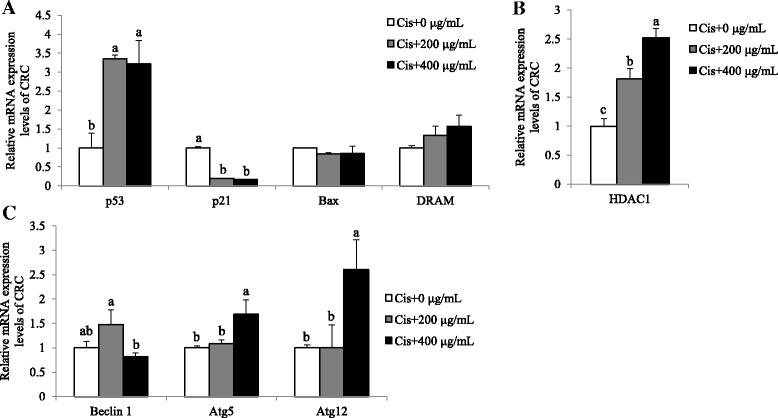
Combination effects of SbE and cisplatin on mRNA expression levels of *p53*, *p21*, *Bax*, *DRAM*, *Beclin 1*, *Atg5*, and *Atg12* in CRC. After cells were exposed 200 ~ 400 μg/mL of SbE and 30 μM of cisplatin for 24 h, mRNA expression levels of (**a**) *p53*, *p21*, *Bax*, *DRAM*, (**b**) *HDAC1*, (**c**) *Beclin 1*, *Atg5*, and *Atg12* in CRC were quantified by qPCR. All mRNA expression levels were normalized against *TBP*. Each value is the mean ± SD. *p* < 0.05 was taken to define statistical significance. One-way ANOVA followed by Duncan post hoc test. Cis; 30 μM of cisplatin

We further examined a possible mechanism underlying the combination effect. We tested whether the cell death upon the SbE treatment combined with cisplatin is mediated by either p53 pathway and/or autophagic pathway in the condition of 200 ~ 400 μg/mL of SbE and 30 μM of cisplatin treatment for 24 h. In CRC, mRNA expression level of *p53* significantly increased in a SbE (*p* < 0.05), while that of *p21* decreased (*p* < 0.001; Fig. 7a). In addition, the expression of *Bax* and *DRAM* did not significantly change (Fig. 7a). Surprisingly, the dramatically decreased expression of *p21* was not consistent with the increased expression of *p53,* even though p21 is a well-known transcriptional target of p53. We investigated if the expression of *p21* was regulated by additional factors such as epigenetic modulators. HDAC1 and p53 have been demonstrated as antagonistic regulators at the *p21* locus. mRNA expression levels of *HDAC1* significantly increased upon the combined treatment of SbE and cisplatin (*p* < 0.05; Fig. 7b), restulting in the transcriptional repression of *p21.* Instead**,** we examined mRNA expression levels of autophagy genes such as *Beclin 1*, *Atg5*, and *Atg12*. mRNA expression levels of *Beclin 1* did not change (Fig. 7c) in the combination-treated group, compared with the non-treated group. However, the expression of *Atg5* and *Atg12* increased upon the treatment (*p* < 0.05; Fig. 7c). Taken together, the results demonstrated that the combination therapy of SbE and cisplatin effectively induced cell death even in CRC via the autophagy pathway independent of Beclin 1.

## Discussion

In this study, we investigated the effect and the possible molecular mechanism of SbE on cisplatin resistance of ovarian cancer by three steps. First of all, we validated the cell models whether CSC and CRC showed different sensitivity against cisplatin, as expected. Next, we examined whether SbE induced cell death in ovarian cancer and what molecular mechanism was involved. Lastly, we demonstrated the effects of combined treatment with SbE and cisplatin on cisplatin resistance in ovarian cancer.

We found that cisplatin induced cell death in CSC in a dose-dependent manner based on up-regulation of genes associating apoptosis pathway such as *p53*, *p21*, and *Bax* expression. As the cellular response to DNA damage, the tumor suppressor and transcription factor p53 and its targets, cyclin-dependent kinase inhibitor p21 and pro-apoptotic Bax, play important roles in apoptosis [25]. It is also critical to modulate the chemosensitivity of tumors by controlling cell death [26]. However, CRC showed cisplatin resistance even though the expression of pro-apoptotic protein *p21* was up-regulated. One possibility is due to decreased *DRAM* mRNA expression level. A previous study showed that DRAM is a major component of p53-induced apoptosis [16]. Its knockdown decreased percentage of apoptosis even though mRNA expression levels of *p53* and *p21* increased in TetOn-p53 cells and RKO cells. Our results demonstarated that cisplatin treatment led to down-regulated expression of the *DRAM* gene in CRC, while it did not in CSC. Therefore, p53-mediated apoptotic pathway seemed to be blocked due to the decreased expression of *DRAM* in the cisplatin resistant cells.

The cisplatin-mediated cell death was not dependent on autophagy. The mRNA expression of *Atg5* levels was significantly decreased in both CSC and CRC. Because Atg12-Atg5 conjugate is an essential factor of autophagy, concordant expression of *Atg12* and *Atg5* expression is induced during autophagy. In a previous study, the Atg5 mutant was unable to generate the Atg12-Atg5 conjugate, resulting in the decrease of autophagic activity rapidly compared with the wild type [27].

Recently, the flavonoid baicalin enriched in herbal medicines including SbE [28] has reported to have anti-cancer properties in vivo and in vitro [21, 29]. We showed that an inhibitory effect of SbE as the extract on cell viability was greater than that of baicalin as a single compound in ovarian cancer cells. SbE induced cell death, depending on its concentration, by inducing expression of apoptosis genes, such as *p53*, *p21*, and *Bax,* and autophagy genes such as *Atg5* and *Atg12* in CSC, but not in CRC. The results showed that CRC had lower efficiency against a single treatment of cisplatin or SbE, compared to CSC. However, combination treatment of SbE and cisplatin enhanced anti-cancer effects via induction of cell death. When 400 μg/mL of SbE and 30 μM of cisplatin were treated at the same time, cell viability was no difference between CSC and CRC. Intriguingly, the combination treatment did not fully induce p53-mediatic apoptotic pathway. Even though *p53* expression increased, its target gene *p21* expression decreased. One of the possibilities is the up-regulation of HDAC1, an epigenetic modulator. Recently, epigenetic mechanism has been highlighted in cancer field. Epigenetic modulation contributes to cancer development, progress, and treatment, since it regulates expression of oncogenes and tumor suppressor genes by altering chromatin structure [30]. Chemical modifications on DNA and core histones, the octamer of proteins wrapping DNA, make chromatin condensate or unwind without altering DNA sequence [31]. Histone acetylation, an example of histone modifications, is a process that an acetyl group is bound to histone resulting in neutralizing DNA charge then forming euchromatin. Euchromatic structure allows a gene to be up-regulated by loosely unfolding nucleosomes and making transcriptional factors easily access to DNA [32]. The acetylated histones are accomplished by histone acetyltransferases (HATs) and the acetyl group is detached by histone deacetylases (HDACs) from histones. A previous study showed that p53 and the epigenetic regulator HDAC1 are antagonistic regulators of the p21 [33]. p53 transcriptionally activates p21 through binding to the transcription factor Sp1 in the activation of the p21 promoter. On the other hand, HDAC1 transcriptionally represses *p21* gene expression by blocking the interaction between p53 and Sp1. In CRC, the combination treatment repressed *p21* gene expression through HDAC1 activation, resulting in inactivation of apoptosis. However, further experiments are needed to verify whether up-regulation of *HDAC1* expression resulted from the combined treatment alters histone acetylation levels, in particular at the *p21* locus. Nevertheless, the combination of SbE and cisplatin induced cell death via autophagy in CRC, showing effectiveness to overcome the resistance. It was mediated by non-canonical Beclin 1*-*independent autophagic cell death based on the increase of *Atg5* and *Atg12* expression, but no change in *Beclin-1* expression. Beclin 1 is related to form a phagophore which is consist of autophagosome [18]. However, Beclin 1*-*independent autophagy has been reported [34, 35]. Resveratrol induced cell death through Beclin 1*-*independent Atg12-Atg5-dependent autophagy [34]. Arsenic trioxide also induced Beclin 1-independent autophagic pathway in ovarian cancer cells. In the arsenic trioxide-treated ovarian cancer cells, Atg5 knockdown reduced autophagy via altering the ratio of LC3-II/LC3-I, which is an indicator of the autophagic progress [36]. In contrast, Beclin 1 knockdown did not alter the ratio of LC3-II/LC3-I [35]. Our data suggest that the combination treatment of SbE and cisplatin produced synergistic anti-cancer effect even in cisplatin resistant ovarian cells. Further analysis is needed to confirm the molecular alterations at protein levels and the extension of anti-cancer effects, i.e. the percentage of apoptotic and/or autophagic cell death.

## Conclusions

Compared with SbE or cisplatin alone, the combination treatment of SbE and cisplatin had strengthened anti-cancer effects in ovarian cancer cells. Although SbE induced cell death in ovarian cancer cells in a dose-dependent manner, the efficiency was significantly lower in CRC, compared to CSC. However, the combination treatment with cisplatin led to the effect on CRC, as similar as on CSC, suggesting the effectiveness of the combined treatment over chemoresistance. The combination treatment in CRC induced autophagy by up-regulated expression of *Atg5* and *Atg12*. It was different from the fact that SbE as a single treatment failed to induce apoptosis via p53 or autophagic pathways in CRC. Taken together, the results demonstrated that the combination treatment of SbE and cisplatin had a synergistic effect by inducing Beclin 1-independent autophagy in CRC. The findings suggest that the combination of SbE and cisplatin may be useful for a potential chemotherapy to treat ovarian cancer.

## Abbreviations

3 MTT: 3-[4,5-dimethylthiazol-2-yl]-2,5-diphenyltetrazoliumbromide
Atg: Autophagy-related protein
CRC: Cisplatin resistant human ovarian cancer cell line
CSC: Cisplatin sensitive human ovarian cancer cell line
DMSO: Dimethyl sulfoxide
DRAM: Damage-regulated autophagy modulator
FBS: Fetal bovine serum
HATs: Histone acetyltransferases
HDACs: Histone deacetylases
LC3: Microtubule-associated protein light chain
SbE: Extract of *Scutellaria baicalensis*
